# Nonsurgical Facial Rejuvenation with Neuromodulators and Soft Tissue Fillers: Evaluating Outcomes of a Longitudinal Resident-Run Clinic

**DOI:** 10.1093/asjof/ojaf018.020

**Published:** 2025-05-13

**Authors:** Kaylee Scott, Ashraf A Patel, Sydney Somers, Aaron Dadzie, Cori A Agarwal, Renato Saltz, Courtney Crombie

**Affiliations:** University of Utah, Salt Lake City, UT; University of Utah, Salt Lake City, UT; University of Utah, Salt Lake City, UT; University of Utah, Salt Lake City, UT; University of Utah, Salt Lake City, UT; University of Utah, Salt Lake City, UT; University of Utah, Salt Lake City, UT

## Abstract

**Goals/Purpose:**

Resident-run clinics (RRCs) have emerged as a vital part of aesthetic plastic surgery training. Approximately 60-70% of plastic surgery residency programs have dedicated RRCs, which play a crucial role in enhancing resident cosmetic surgery exposure and autonomy. At certain institutions, RRCs have been developed to increase resident exposure to nonsurgical methods of facial rejuvenation, either as a separate entity or engrained within a resident cosmetic surgery clinic. These clinics have an emphasis on neuromodulators, soft tissue fillers, and at some institutions, lasers and peels. At The University of Utah, a RRC offering resident-administered neuromodulators and soft tissue fillers occurs on a weekly basis, and integrates residents of all years with a focus on graduated autonomy. PGY-1 and PGY-2 residents perform proctored neuromodulator and soft tissue filler injections prior to autonomously participating in the clinic as a PGY-3. These proctored injections often occur at biannual injection training sessions under either chief resident or attending supervision, but may also occur in the RRC. Though similar training models have been described in the literature, outcomes of these clinics have not been consistently described. The purpose of this study is to describe patient demographics, injection characteristics, resident qualities, and outcomes of this longitudinal clinic.

**Methods/Technique:**

A retrospective chart review of all patients who attended The University of Utah Plastic and Reconstructive Surgery RRC from January 2017 to July 2024 was performed. Patient demographics, type and location of injectable received (neuromodulator vs. soft tissue filler), revision rates, patient retention rates, rates of conversion to cosmetic consultation, and post-graduate year characteristics were then evaluated.

**Results/Complications:**

A total of 380 patients were identified. The majority of patients were female (92.1%) with a mean age of 41. Of these patients, 42% had never received a neuromodulator or filler injection before, 16% had been injected by another university provider, and 40% had received injections from an outside provider. There was a total of 1,291 patient encounters, with an average of 3.3 visits per patient. A total of 37 residents rotated in the clinic during the study period. Overall, 2.0% of encounters were performed by PGY-1s, 4.7% by PGY-2s, 8.9% by PGY-3s, 15.2% by PGY-4s, 15.9% by PGY-5s, 20.2% by PGY-6s, 17.0% by PGY-7s, and 16.9% by PGY-9s. Neuromodulator injections represented 53.4% of encounters, filler injections represented 23% of encounters, and combined neuromodulator and filler injections represented 23.5% of encounters. The most common neuromodulator injection sites were corrugators and procerus (86.9%), frontalis (87.4%), and orbicularis oculi (66.3%). The most common filler injection sites were the lips (75.5%), nasolabial folds (20.6%), and cheeks (18.1%). There were 72 encounters for revisions, resulting in a 5.5% revision rate. These revisions occurred in patients receiving neuromodulators (47.2%), filler (43%), and neuromodulators and filler combined (8.3%). The most common reason for neuromodulator and filler correction was asymmetry (47.5% and 51.3%). Overall, 272 patients returned to clinic for subsequent injections following their first visit, resulting in a retention rate of 71.5%. A total of 4.2% of patients scheduled a surgical consult with a chief resident, 5% scheduled a surgical consult with an attending, and 5.5% scheduled a consult with an aesthetician following an injection encounter. Overall, the conversion rate from cosmetic injection to surgery was 5.8%. There was a low complication rate overall (0.8%), with 1 incidence of intravascular injection managed with hyaluronidase, 1 incidence of self-limited eyelid ptosis, and 1 incidence of infection that required incision and drainage and antibiotics.

**Conclusion:**

The RRC was found to integrate residents of all training levels, with an emphasis on residents in year PGY-3 and beyond. The demographics of the patients in this clinic closely reflects what have been described by ASPS nationally for injectable use. Despite the wide range in resident training level, the rate of revision procedures was relatively low (5.5%), as well as complication rates overall (0.8%). Additionally, retention rates were high (71.5%), and the rate of patient conversion to cosmetic surgery was similar to that described in other studies. The RRC was found to be a safe and valuable training tool to provide longitudinal exposure to nonsurgical facial rejuvenation. Additionally, this data suggests that RRCs may have similar outcomes and productivity to other cosmetic injection clinics. Additional studies evaluating patient satisfaction, resident satisfaction, and cost-effectiveness may help further evaluate the efficacy of this clinic.

